# A case report of primary hyperparathyroidism due to a giant substernal parathyroid adenoma managed using a neck incision approach

**DOI:** 10.1097/MD.0000000000046659

**Published:** 2025-12-26

**Authors:** Mengya Shi, Songxiang Wang, Yuancong Jiang, Hanjin Wu

**Affiliations:** a Department of Geriatrics, Shaoxing People’s Hospital, Shaoxing, China; b School of Medicine, Shaoxing University, Shaoxing, Zhejiang, P.R. China; c Department of Breast and Thyroid Surgery, Shaoxing People’s Hospital, Shaoxing, China.

**Keywords:** faster postoperative recovery, parathyroid adenoma, primary hyperparathyroidism, substernal tumor, trans neck surgery

## Abstract

**Rationale:**

Primary hyperparathyroidism is an endocrine disease primarily caused by a single benign parathyroid adenoma. It is characterized by symptoms such as hypercalcemia, hypophosphatemia, kidney stones, and bone destruction. Parathyroid adenomas are generally located on the posterior aspect of the thyroid gland, while giant parathyroid adenomas in the anterior superior mediastinum are relatively rare. Typically, benign anterior superior mediastinal tumors are managed through sternotomy or thoracoscopic surgery, which can be associated with significant trauma or complications.

**Patient concerns:**

We present a case of a patient with hypercalcemia and hyperparathyroidism. Biochemical analysis showed that the serum calcium level was 2.72 mmol/L and the parathyroid hormone level was 1050 pg/mL. Physical examination, ultrasound, and CT scans revealed a lesion in the right anterior superior mediastinum, suggesting it originated from the right inferior parathyroid gland.

**Diagnoses:**

Based on the physical examination, biochemical, and imaging findings, the patient was preliminarily diagnosed with a parathyroid tumor in the right anterior superior mediastinum.

**Interventions:**

The patient underwent successful resection of a giant anterior superior mediastinal parathyroid adenoma via a neck incision.

**Outcomes:**

At the 1-year follow-up, the patient was alive and had normal voice function. There was no abnormal increase in serum calcium or parathyroid hormone levels.

**Lessons:**

For certain benign anterior superior mediastinal tumors, a neck incision is recommended to minimize surgical trauma and promote faster postoperative recovery.

## 1. Introduction

Primary hyperparathyroidism (PHPT) is a common endocrine disorder characterized by elevated serum calcium and parathyroid hormone levels.^[1]^ Approximately 80% to 85% of PHPT cases are attributed to a single benign parathyroid tumor.^[2]^ Surgical removal of the parathyroid adenoma remains one of the most effective treatments for alleviating clinical symptoms and reducing systemic complications. Traditional parathyroid adenomas located in the neck are typically addressed through a neck incision. Nevertheless, approximately 2% to 5% of adenomas present as ectopic, hyperfunctioning mediastinal parathyroid adenomas, posing significant treatment challenges.^[3–5]^ Parathyroid glands are often located in the anterior superior mediastinum, making complete removal through simple neck surgery difficult. Consequently, benign tumors in this region are typically treated using thoracotomy approaches, such as sternal splitting or video-assisted thoracoscopic surgery.^[6]^ Thoracotomy provides excellent exposure to the surgical field under direct vision and facilitates effective bleeding control. However, it is associated with significant trauma and a slower recovery process.^[7]^ Although video-assisted thoracoscopic surgery allows for complex procedures using high-definition, camera-assisted imaging, it remains challenging to visualize the anatomy of the contralateral mediastinum through a unilateral chest wall incision. This limitation may result in incomplete tumor resection or unintended injury to the contralateral phrenic nerve or innominate vein.^[7,8]^ With careful case selection, an open cervical incision may provide a more suitable surgical approach for removing anterior superior mediastinal tumors, offering the benefits of reduced trauma and faster recovery.^[9]^ Although the space behind the sternum is narrow, the anterior superior mediastinum can be adequately visualized by applying firm traction to the sternum. By dissecting from superficial to deep planes, certain benign tumors in the anterior superior mediastinum can be completely removed through a cervical incision, thereby avoiding the need for sternal splitting or transthoracic surgery. Here, we report a case of primary hyperparathyroidism caused by a giant substernal parathyroid adenoma, successfully treated with a transcervical approach. Based on the surgical experience from this case, we believe that select benign tumors of the anterior superior mediastinum can be effectively treated through a cervical incision, offering the advantages of reduced surgical trauma and faster postoperative recovery.

## 2. Case presentation

**Figure 1.**
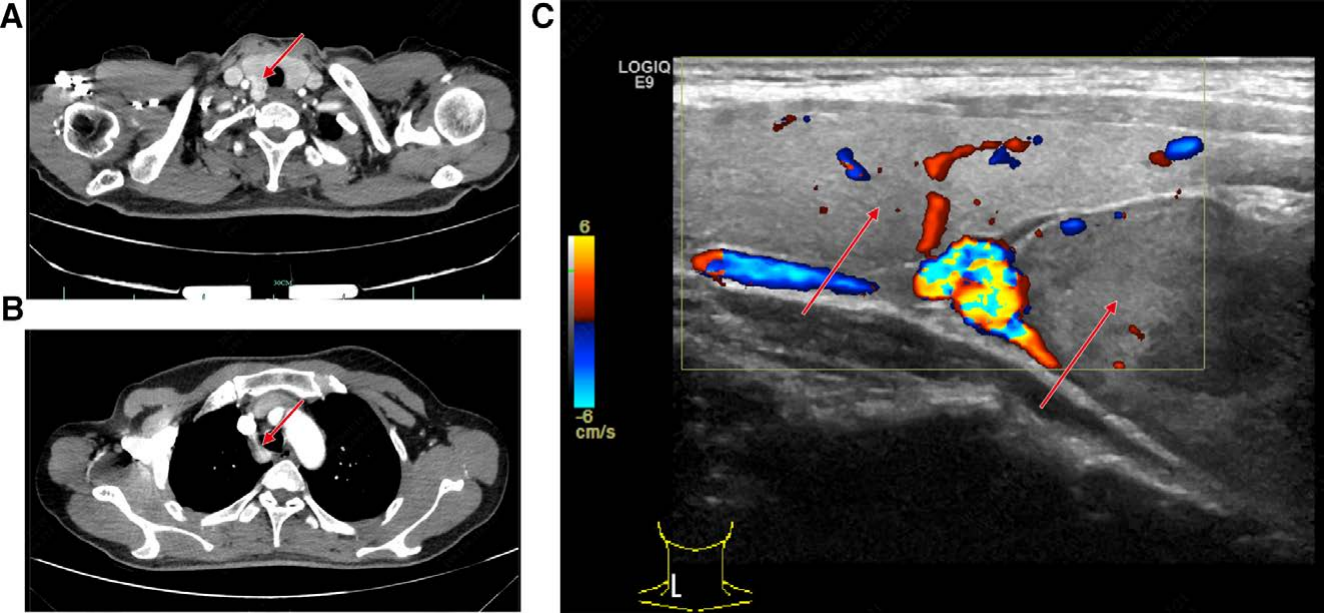
(A) CT scan images of the right anterior superior mediastinal parathyroid adenoma. The arrow indicates the upper edge of the parathyroid adenoma located behind the right thyroid gland. (B) CT scan images show the lower edge of the right anterior superior mediastinal parathyroid adenoma at the level of the aortic arch, as indicated by the arrow. (C) Ultrasound examination images of the parathyroid glands in the neck. The left arrow points to the right thyroid gland, and the right arrow points to the right parathyroid adenoma, located near the lower edge of the thyroid gland.

**Figure 2.**
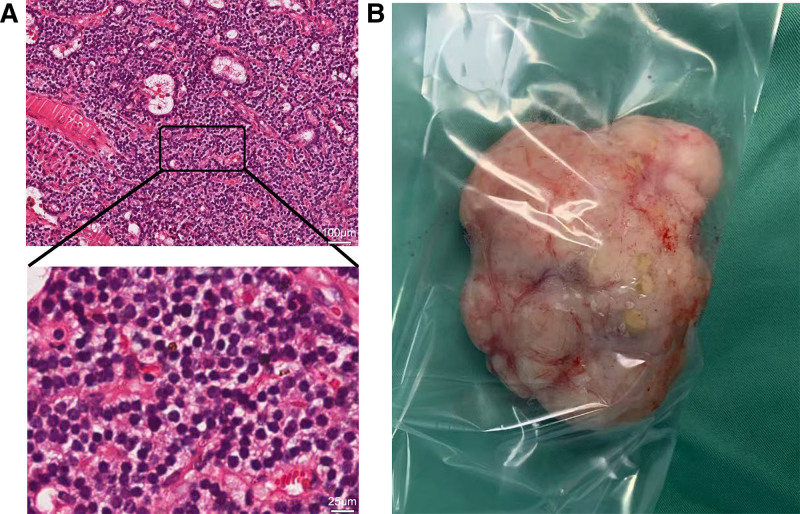
(A) High-resolution hematoxylin-eosin staining images of the right anterior superior mediastinal parathyroid adenoma. Magnification, 100×; scale bar, 100 μm and magnification, 400×; scale bar, 25 μm. (B) Gross images of the parathyroid adenoma.

A 42-year-old female was admitted to the hospital in November 2021 with a painless mass in the right superior and anterior mediastinum. The patient did not report any symptoms, but physical examination revealed that the upper edge of the tumor could be palpated behind the lower pole of the right thyroid during swallowing. The mass had a smooth surface, a medium-soft texture, and no tenderness. The tumor could not be palpated in its entirety but moved up and down with swallowing. A chest computed tomography (CT) scan showed a lesion occupying the right anterior superior mediastinum. An enhanced chest CT revealed a round soft tissue density measuring approximately 6.0 × 3.1 × 2.8 cm in the right anterior mediastinum. The lesion had a well-defined edge and showed significant enhancement after contrast administration, with its upper margin connected to the right thyroid gland (Fig. 1A and B). Parathyroid ultrasound identified a hypoechoic mass below the inferior pole of the right thyroid gland, suggesting it originated from the right inferior parathyroid gland (Fig. 1C). However, no similar features were observed in the other parathyroid glands on either side. Biochemical analysis revealed a serum calcium level of 2.72 mmol/L and a parathyroid hormone level of 1050 pg/mL. Based on these findings, the patient was preliminarily diagnosed with a parathyroid tumor in the right anterior superior mediastinum. Given the tumor’s large size and its base extending to the level of the aortic arch, the most straightforward surgical approaches would typically involve trans-sternotomy or thoracoscopic surgery. Nevertheless, these methods are more invasive and associated with slower postoperative recovery compared to a trans-cervical incision approach. After thorough communication between the medical team and the patient, it was decided to attempt the removal of the parathyroid tumor in the anterior superior mediastinum through a neck incision, with trans-sternotomy or thoracoscopic surgery as backup options. After successful anesthesia, the patient was placed in the supine position with a pillow under the shoulders and back. The surgical field was routinely disinfected and covered with a drape. The procedure began with a low collar-like incision on the neck. The right recurrent laryngeal nerve served as a key anatomical marker behind the sternum. The dissection was carried out using a combination of blunt and sharp techniques, proceeding downward along the deep fascial plane of the neck. A free muscle flap was made from the thyroid cartilage to the sternal notch. The white line of the neck was incised, the anterior cervical muscles were separated, the right thyroid body was freed, and it was pulled upward to expose the mass behind and below it. Most of the mass was located behind the sternum and was considered to be a right ectopic retrosternal parathyroid adenoma. The adhesions between the right recurrent laryngeal nerve and the right parathyroid gland were carefully dissected, extending downward to the origin of the right brachiocephalic trunk behind the sternum. The surrounding blood vessels were coagulated with an ultrasonic scalpel, and the parathyroid tissue at the top was secured with a vascular clamp. The giant anterior superior mediastinal parathyroid adenoma was gradually pulled into the neck and successfully removed. The right recurrent laryngeal nerve was observed migrating along the surface of the mass, with noticeable adhesions between the nerve and the tumor. The substernal parathyroid adenoma was excised through the cervical approach. The procedure was performed for rapid resection, with care taken to protect the recurrent laryngeal nerve. After hemostasis and lung inflation, no bleeding or air leakage was observed. A drainage tube was placed in the wound, connected to negative suction, and the wound was sutured continuously with 5-0 absorbable sutures. The procedure was smooth, and bleeding was minimal. Fifteen minutes after the removal, parathyroid hormone levels were measured at 70 pg/mL. Intraoperative frozen section analysis confirmed the diagnosis of a right anterior superior mediastinal parathyroid adenoma. Representative hematoxylin-eosin staining and Gross images of the parathyroid adenoma are depicted in Figure 2. Slight hoarseness occurred after the operation due to repeated traction on the right recurrent laryngeal nerve during the procedure. On the third day postoperatively, serum calcium was 1.98 mmol/L, and parathyroid hormone was 53 pg/mL. Postoperative histopathology confirmed the presence of a parathyroid adenoma in the right anterior superior mediastinum, with some cells showing active proliferation. Immunohistochemical results were as follows: CgA (+), CK19 (-), TTF-1 (-), CD10 (-), and Ki-67 (+ < 1%). At the 1-year follow-up, the patient was alive and had normal voice function. There was no abnormal increase in serum calcium or parathyroid hormone levels. Future follow-up appointments were scheduled annually.

## 3. Discussion

PHPT and parathyroid adenoma are relatively rare in China, and the occurrence of PHPT due to giant substernal parathyroid adenoma is even less common.^[10,11]^ Most benign tumors of the anterior superior mediastinum are thyroid tumors, parathyroid tumors, lymphangiomas, and thymomas, to name a few.^[11,12]^ The most used surgical approaches are thoracotomy with sternal splitting or video-assisted thoracoscopic surgery. Some experts also suggest that a neck incision combined with thoracoscopic surgery can be applied in select cases.^[13]^ Since benign tumors of the anterior superior mediastinum do not typically infiltrate important structures such as peripheral blood vessels and nerves, direct resection through a cervical incision is a viable surgical option following careful assessment.^[9,14]^

Parathyroid tumors are common benign lesions found in the anterior superior mediastinum. During embryonic development, if the inferior parathyroid embryonic primordium fails to separate from the thymic embryonic primordium after descending to the lower thyroid pole, it may descend into the mediastinum along with the thymic primordium. Some of these cells may undergo neoplastic transformation and hyperplasia, forming parathyroid tumors in the mediastinum.^[15]^ It has been reported that anterior superior mediastinal parathyroid tumors are generally small, weighing between 70 mg and 1 g, and rarely exceeding 20 g.^[16]^ Some anterior superior mediastinal parathyroid tumors are so small that even a 99mTc-MIBI dual-phase scan may fail to detect them, leading to missed diagnoses.^[17]^ However, there have also been reports of parathyroid tumors in the upper right mediastinum reaching sizes of 8 × 3 × 3 cm, which can pose significant challenges for surgical management.^[18]^ In the present case, preoperative CT imaging revealed a tumor size of 6.0 × 3.1 × 2.8 cm with a longitudinal shuttle shape, classifying it as a relatively large anterior superior mediastinal parathyroid tumor. Preoperative ultrasound and CT also indicated that the upper edge of the tumor was very close to the right inferior thyroid gland. During the physical examination, the movable upper edge of the tumor could be palpated in the neck, providing the clinicians with the opportunity to remove the anterior superior mediastinal parathyroid tumor through a neck incision.

Surgery is the primary treatment for large anterior superior mediastinal parathyroid tumors. Based on the surgical experience with this patient and a review of the relevant literature, we believe that some benign tumors of the anterior superior mediastinum can be effectively treated through a cervical incision. This approach offers the benefits of reduced surgical trauma and a quicker recovery postoperatively. To adopt this surgical approach, patients should meet the following criteria: Preoperative examinations must indicate that the tumor is benign and located in the anterior superior mediastinum, with no infiltration of important structures such as peripheral blood vessels and nerves. The tumor should originate from the neck or thyroid gland, and its upper edge should be palpable and movable in the neck during physical examination. CT and other imaging studies should reveal that the tumor has an isolated, longitudinal, round shape with clear boundaries. The tumor should be centered, not protruding into the thoracic cavities on either side, and the lower edge should not extend below the level of the aortic arch. The patient should have no lower neck or mediastinal surgery history.

## 4. Conclusion

In conclusion, our report suggests that resection of giant parathyroid adenomas in the anterior superior mediastinum can be successfully performed via a neck incision. Although the space behind the sternum is narrow, it is possible to achieve sufficient exposure of the anterior superior mediastinum by pulling the sternum. By progressively separating from the shallow to the deeper layers, some benign anterior superior mediastinal tumors can be completely removed through the neck, thus avoiding the need for sternotomy or transthoracic surgery.

## Acknowledgments

We thank the Home for Researchers editorial team (www.home-for-researchers.com) for the language editing service.

## Author contributions

**Supervision:** Yuancong Jiang, Hanjin Wu.

**Writing – original draft:** Mengya Shi, Yuancong Jiang.

**Writing – review & editing:** Songxiang Wang, Hanjin Wu.
